# Feedback between bottom-up and top-down control of stream biofilm mediated through eutrophication effects on grazer growth

**DOI:** 10.1038/s41598-021-00856-9

**Published:** 2021-11-03

**Authors:** Alessandra Iannino, Patrick Fink, Markus Weitere

**Affiliations:** 1 Department of River Ecology, Helmholtz Centre for Environmental Research—UFZ, Brückstrasse 3a, 39114 Magdeburg, Germany; 2 Workgroup Aquatic Chemical Ecology, University of Cologne, Zülpicherstrasse 47b, 50674 Cologne, Germany; 3 Department of Aquatic Ecosystem Analysis and Management, Helmholtz Centre for Environmental Research—UFZ, Brückstrasse 3a, 39114 Magdeburg, Germany

**Keywords:** Ecology, Freshwater ecology

## Abstract

Algal biofilms in streams are simultaneously controlled by light and nutrient availability (bottom-up control) and by grazing activity (top-down control). In addition to promoting algal growth, light and nutrients also determine the nutritional quality of algae for grazers. While short-term experiments have shown that grazers increase consumption rates of nutrient-poor algae due to compensatory feeding, nutrient limitation in the long run can constrain grazer growth and hence limit the strength of grazing activity. In this study, we tested the effects of light and phosphorus availability on grazer growth and thus on the long-term control of algal biomass. At the end of the experiment, algal biomass was significantly affected by light, phosphorus and grazing, but the interactive effects of the three factors significantly changed over time. At both high light and phosphorus supply, grazing did not initially reduce algal biomass, but the effect of grazing became stronger in the final three weeks of the experiment. Snail growth was enhanced by light, rather than phosphorus, suggesting that algal quantity rather than quality was the main limiting factor for grazer growth. Our results highlight the role of feedback effects and the importance of long-term experiments in the study of foodweb interactions.

## Introduction

The abundance of algal biofilms (periphyton) in stream ecosystems is determined both by the availability of resources such as light and nutrients (bottom-up control) and by the grazing activity of herbivores (top-down control). Algal biomass increases with light and nutrient availability, while it is decreased by grazing^1,2^. However, bottom-up and top-down control can strongly interact with each other, and their relative strength over one another may vary under different environmental conditions^3,4^. In particular, variations in light and nutrient supply may alter the nutritional quality of periphyton for grazers and hence affect grazer growth and behaviour. Periphyton nutritional quality for grazers is determined by the algal content of phosphorus (P) and nitrogen (N) relative to carbon (C), i.e. molar C:P and C:N stoichiometric ratios^5,6^. High C:P and C:N ratios in algae can severely constrain herbivore growth and reproduction rates^7–9^. Algal C:P and C:N ratios increase with light availability, as photosynthetic activity leads to the production of organic carbon, whereas they decrease as nutrient supply increases^10,11^.

Both light and nutrient availability in streams have been altered in the past decades as a consequence of human disturbances. Agricultural runoffs and industrial activities have been releasing excessive amounts of nutrients into aquatic environments, leading to eutrophication^12^, while the clearcutting of riparian vegetation has resulted in increased irradiance levels at the streambed^13^. Grazing activity of herbivores has the potential to counteract the enhanced algal growth resulting from increased light and nutrient inputs; however, it is critical to understand how the strength of top-down control is affected by changes in algal nutritional quality related to variations in resource availability. Grazers often adopt behavioural strategies to optimise nutrient intake, such as adjusting consumption rates depending on the relative nutrient content of their food. Low relative content of nutrients results in increased consumption rates, a behaviour known as compensatory feeding^8,14^. Therefore, top-down control may be stronger at low nutrient supply, and nutrient enrichment may result in increased algal biomass not only directly, but also indirectly by reducing food consumption rates of grazers, as we have demonstrated in a previous study^15^.

However, compensatory feeding is only a short-term behavioural response to changes in algal nutritional quality. While compensatory feeding is effective at maintaining a constant soft body stoichiometry in grazers^15^, it comes at an energetic cost and is often not enough to offset the harmful consequences of nutrient limitation for grazers^8,16^. Therefore, in the long run, low nutrient supply could constrain grazer growth and reproduction and hence limit the strength of top-down control. Yet, most periphyton-grazer studies are too short to capture a quantitative response on the consumer biomass level, so changes in the relative strength of top-down control due to grazer growth over extended periods of time are still poorly understood.

In this study, we investigated the long-term effects of phosphorus- and light-driven eutrophication on grazer growth mediated by changes in periphyton C:P stoichiometry, and the resulting feedback on periphyton top-down control. Our model organism was the rheophilic, herbivorous gastropod *Ancylus fluviatilis*, which is widespread in European rivers^17,18^. We performed a mesocosm experiment in which natural periphyton was grown for eight weeks in a fully factorial combination of phosphorus (ambient concentrations versus phosphorus addition), light (low versus high light), and grazing (presence versus absence of grazers). The following hypotheses were tested: (1) Periphyton quantity would increase with both light and phosphorus availability, whereas periphyton C:P stoichiometry would increase with light and decrease with phosphorus supply; (2) Grazer growth would be enhanced by high periphyton quantity and low algal C:P ratios, leading to the highest grazer growth rates at high phosphorus and low light availability; (3) Over the snail growing season, the enhanced snail growth associated with low algal C:P ratios would result in the strongest top-down control of periphyton biomass, as larger grazers would increase their consumption rates. Finally, as the community composition of periphyton can significantly influence its C:P stoichiometry^19,20^, we also determined the relative abundance of algal groups in periphyton in the different treatments via pigment analysis^21^, to check whether they would be affected by phosphorus and light, and in turn potentially impact grazer growth and feeding behaviour. The experiment was performed in the summer and coincided with the growing season of *A. fluviatilis*, which has an annual life cycle^18,22^.

## Results

### Periphyton properties

**Figure 1:**
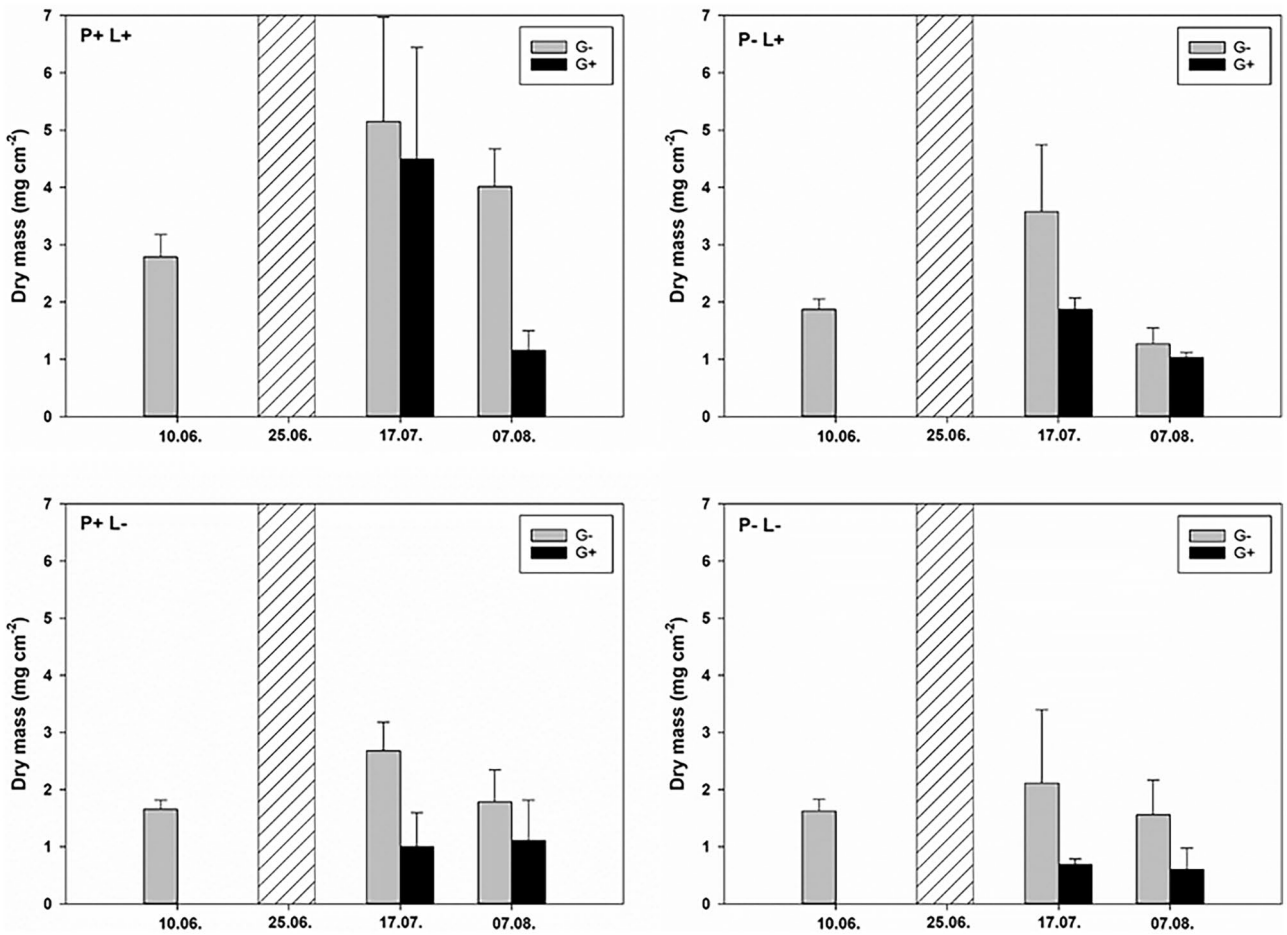
Interactive effects of phosphorus, light and grazing on periphyton biomass over time. Graphs depict periphyton dry mass over time at the beginning and during the grazing phase, at high phosphorus (P+) and low phosphorus supply (P−), high light (L+) and low light availability (L−), with grazers (black bars, G+) and without grazers (grey bars, G−). Phosphorus and light significantly increased periphyton dry mass at the beginning of the grazing phase (two-way ANOVA, Table 1), while time significantly interacted with grazing and phosphorus × light to determine periphyton dry mass during the grazing phase (linear mixed effects model, Table 2).

**Figure 2:**
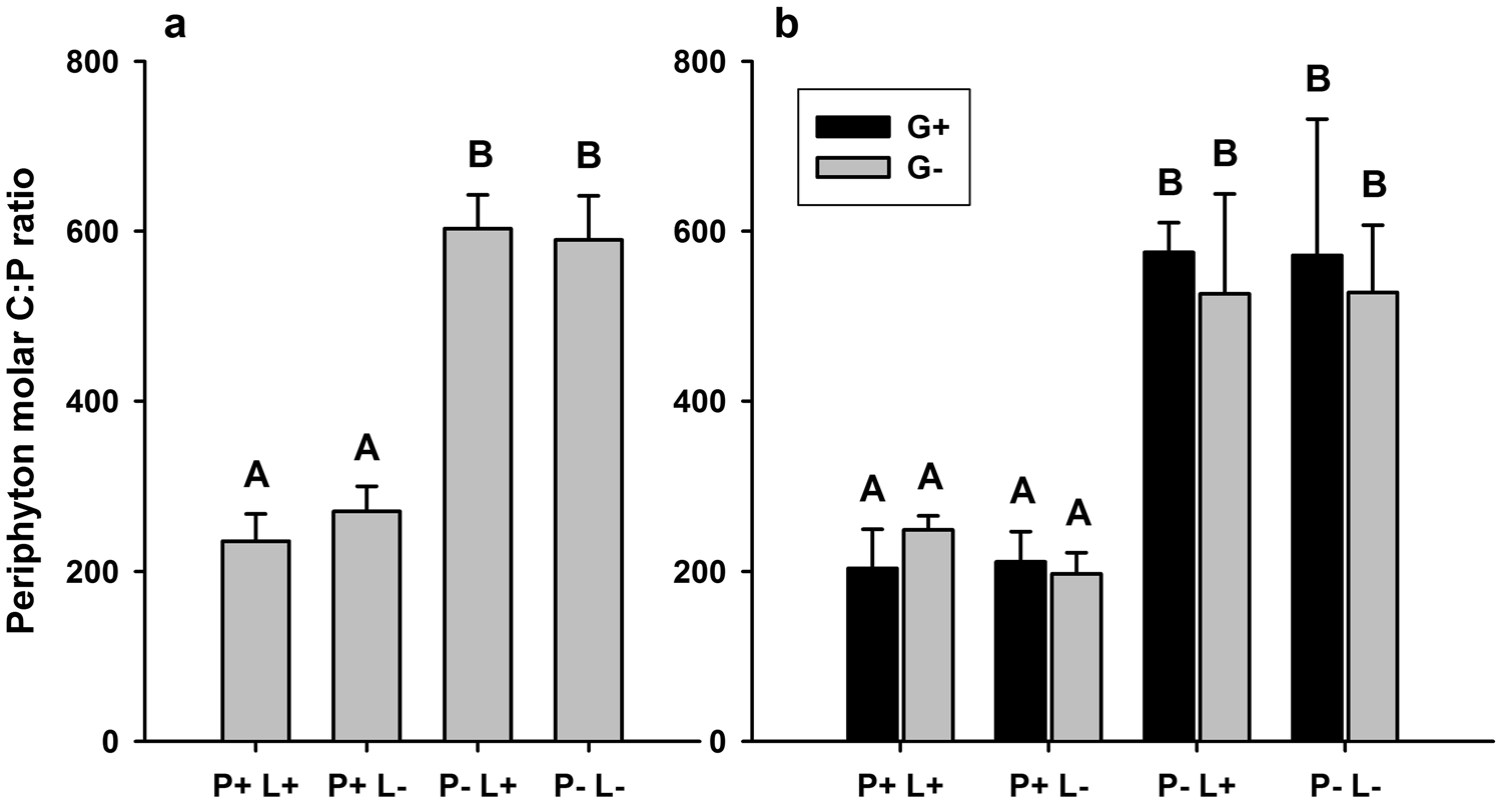
Interactive effects of phosphorus, light and grazing on periphyton C:P stoichiometry. Graphs depict periphyton molar C:P ratio at the beginning (10 June, **a**) and end (7 August, **b**) of the grazing phase at high phosphorus (P+) and low phosphorus supply (P−), high light (L+) and low light availability (L−) and in the presence and absence of grazers (G+ and G−, respectively). Values are mean ± SD of n = 6 (**a**) and n = 3 (**b**) replicate flumes. Phosphorus addition significantly decreased C:P ratios both at the beginning (two-way ANOVA; *F*_1,20_ = 457.01, *p* < 0.001) and at the end of the grazing phase (three-way ANOVA; *F*_1,16_ = 104.64, *p* < 0.001), whereas light and grazing had no effect. Different letters in each panel indicate significant differences between treatments (Tukey’s HSD post-hoc test).

At the beginning of the grazing phase, periphyton dry mass significantly increased as a result of an interaction between phosphorus and light (two-way ANOVA; Table 1; Fig. 1). During the grazing phase, periphyton dry mass was significantly affected by an interaction between time and grazing, as well as between time, phosphorus and light (linear mixed effects model; Table 2; Fig. 2). At high phosphorus and light availability, grazing had a negative effect on periphyton dry mass only in the last three weeks of the grazing phase, i.e. after 17 July (Fig. 1). Without phosphorus addition and/or increased light availability, on the other hand, grazing reduced periphyton dry mass already before 17 July (Fig. 1).

**Table 1:** Results of two-way ANOVAs on the effects of phosphorus and light on periphyton dry mass, molar C:P ratio and diatom percentage contribution to total chlorophyll *a* at the beginning of the grazing phase.

	Dry mass (mg cm^2^)		Periphyton C:P		Diatom %	
	*F*_*1,20*_	*p*	*F*_*1,20*_	*p*	*F*_*1,20*_	*p*
Phosphorus	20.84	**< 0.001**	457.01	**< 0.001**	21.86	**< 0.001**
Light	43.86	**< 0.001**	0.45	0.5	4.41	**0.048**
Phosphorus × light	18.25	**< 0.001**	2.27	0.1	14.40	**0.001**

Significant effects (*p* < 0.05) are highlighted in bold.

**Table 2:** Results of linear mixed effects model on the effects of phosphorus, light and grazing over time on periphyton dry mass.

	d.f	*F*	*p*
Phosphorus	1	12.23	**0.001**
Light	1	16.24	**< 0.001**
Grazing	1	0.79	0.38
Time × phosphorus	1	0.11	0.74
Time × light	1	2.92	0.09
Time × grazing	2	7.19	**0.003**
Time × phosphorus × light	2	3.48	**0.04**
Time × phosphorus × light × grazing	6	1.48	0.21

Significant effects (*p* < 0.05) are highlighted in bold.

**Figure 3:**
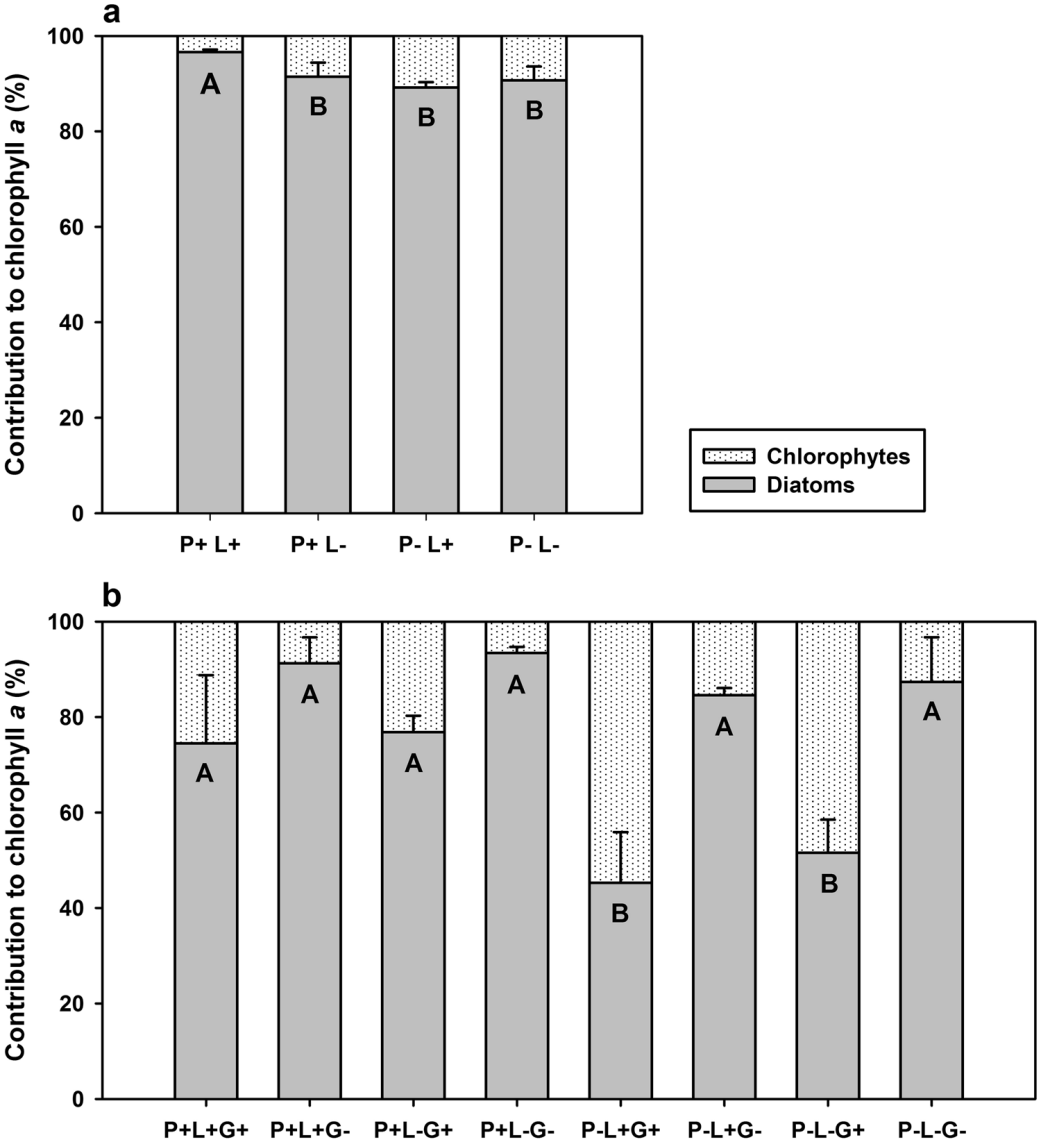
Interactive effects of phosphorus, light and grazing on periphyton taxonomic composition. Graphs depict periphyton relative abundance of diatoms and chlorophytes, measured as percentage contribution to total chlorophyll *a* at the beginning (10 June, **a**) and end (7 August, **b**) of the grazing phase at high phosphorus (P+) and low phosphorus supply (P−), high light (L+) and low light availability (L−) and in the presence and absence of grazers (G+ and G−, respectively). Values are mean ±SD of n = 6 (**a**) and n = 3 (**b**) replicate flumes. Diatom proportion was significantly increased by the interactive effects of P addition and light at the beginning of the grazing phase (two-way ANOVA; *F*_1,20_ = 14.40, *p* < 0.001), whereas it was significantly decreased by the interactive effects of low P supply and grazing at the end of the grazing phase (three-way ANOVA; *F*_1,16_ = 10.50, *p* = 0.005). Different letters in each panel indicate significant differences between treatments (Tukey’s HSD post-hoc test).

Periphyton C:P ratio was significantly decreased by phosphorus addition, but it was not affected by light or grazing, both at the beginning (two-way ANOVA; Table 1; Fig. 2a) and at the end of the grazing phase (three-way ANOVA; Table 3; Fig. 2b). At the beginning of the grazing phase, the periphytic community was mainly composed of diatoms in all treatments (> 85%), with phosphorus and light slightly but significantly increasing the proportion of diatoms over chlorophytes (two-way ANOVA; Table 1; Fig. 3a). At the end of the experiment, the algal communities were significantly affected by an interaction between phosphorus and grazing, with the proportion of diatoms significantly lowered by grazing only at low phosphorus supply (three-way ANOVA; Table 3; Fig. 3b). The proportion of diatoms was reduced to approximately 50% at low phosphorus supply in the presence of grazers and was replaced by chlorophytes (Fig. 3b).

**Table 3:** Results of three-way ANOVAs on the effects of phosphorus, light and grazing on periphyton C:P ratio and diatom percentage contribution to total chlorophyll *a* at the end of the grazing phase.

	Periphyton C:P		Diatom %	
	*F*_*1,16*_	*p*	*F*_*1,16*_	*p*
Phosphorus	104.64	**< 0.001**	27.18	**< 0.001**
Light	0.12	0.7	1.11	0.3
Grazing	0.22	0.6	70.93	**< 0.001**
Phosphorus × light	0.11	0.7	0.13	0.7
Phosphorus × grazing	0.89	0.3	10.50	**0.005**
Light × grazing	0.17	0.7	0.08	0.8
Phosphorus × light × grazing	0.24	0.6	0.07	0.8

Significant effects (*p* < 0.05) are highlighted in bold.

### Snail growth

**Figure 4:**
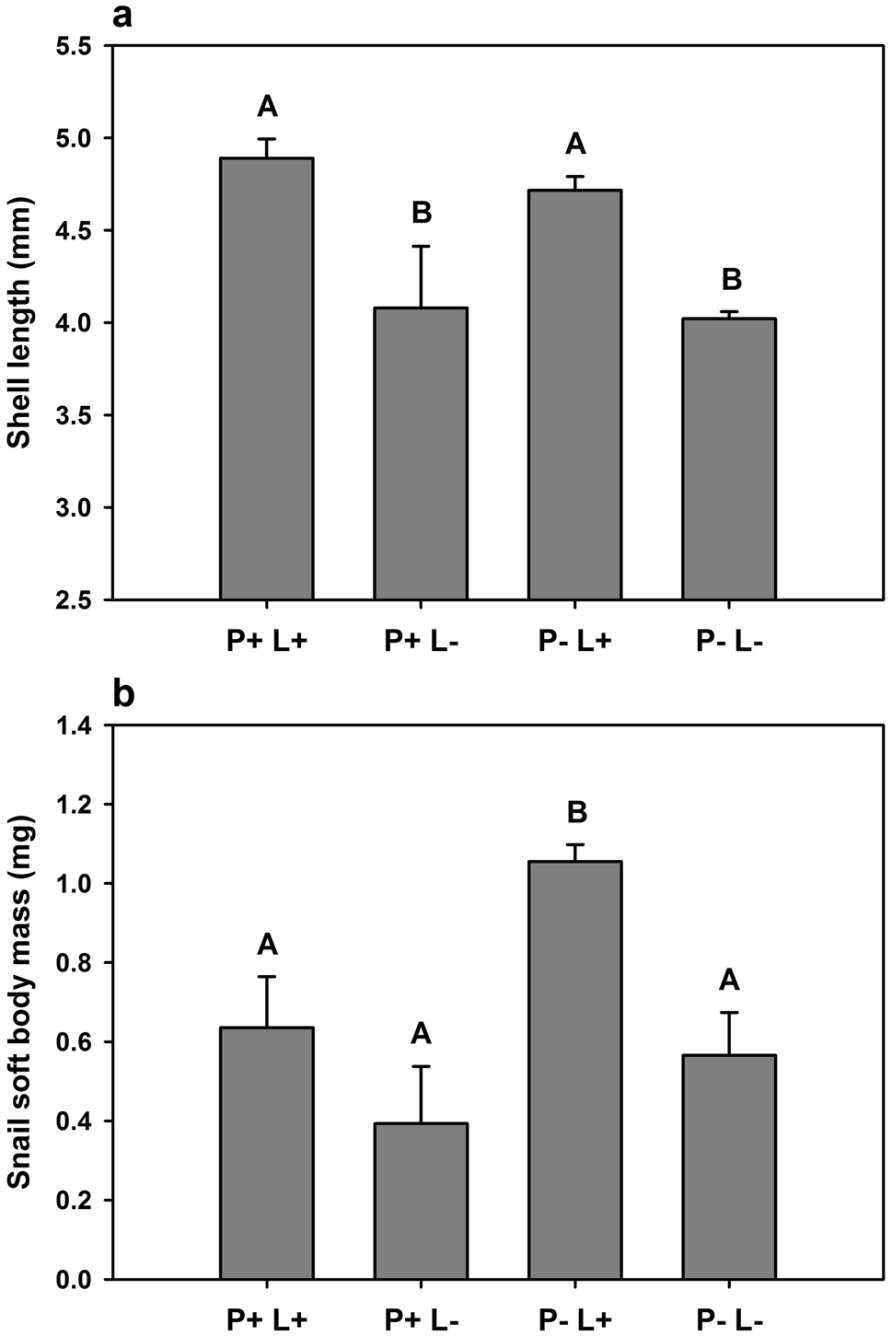
Interactive effects of phosphorus and light on snail growth. Graphs depict the shell length (**a**) and the soft body mass (**b**) of *Ancylus fluviatilis* and at the end of the grazing phase (7 August), at high phosphorus (P+) and low phosphorus supply (P−) and at high light (L+) and low light availability (L−). Values are mean ± SD of n = 3 replicate flumes. Shell length was significantly increased by high light (two-way ANOVA; *F*_1,8_ = 52.69, *p* < 0.001), whereas P had no effect. Soft body mass was significantly increased by both high light (two-way ANOVA; *F*_1,8_ = 31.51, *p* < 0.001) and low P supply (two-way ANOVA; *F*_1,8_ = 20.63, *p* = 0.002). Different letters indicate significant differences between treatments (Tukey’s HSD post-hoc test).

After eight weeks of grazing, snail shell length was significantly increased by high light, but it was not affected by phosphorus (two-way ANOVA; Table 4; Fig. 4a). On the other hand, snail soft body mass was significantly affected by both light and phosphorus (two-way ANOVA; Table 4), with the highest soft body mass observed at high light and low phosphorus availability (Fig. 4b).

**Table 4:** Results of two-way ANOVAs on the effects of phosphorus and light on snail shell length and soft body mass at the end of the grazing phase.

	Shell length		Soft body mass	
	*F*_*1,8*_	*p*	*F*_*1,8*_	*p*
Phosphorus	1.25	0.3	20.63	**0.002**
Light	52.69	**< 0.001**	31.51	**< 0.001**
Phosphorus × light	0.31	0.6	3.60	0.09

Significant effects (*p* < 0.05) are highlighted in bold.

## Discussion

We here demonstrate that, while nutrients, light and grazing all contribute to the control of periphyton biomass, the relative strength of resource availability over grazing pressure changes over time. In the first 5 weeks of the grazing phase, grazing significantly reduced periphyton biomass only when phosphorus and/or light were limiting, and the combined positive effects of high light and phosphorus addition on periphyton growth could offset the negative impact of grazing. However, in the final three weeks of the experiment, grazing pressure became strong enough to significantly decrease periphyton biomass even at high phosphorus and light availability. Therefore, top-down control was initially too weak to counteract the effects of nutrient enrichment and high irradiance on algal growth, but grazers were eventually able to improve their top-down pressure on periphyton biomass in the long run. The relative strength of bottom-up versus top-down control of periphyton was thus strongly dependent on the snail developmental time.

Interestingly, though, the effects of phosphorus and light on periphyton food quality and grazer growth did not support our initial hypotheses. While both light and phosphorus supply increased periphyton biomass, only phosphorus supply affected algal C:P ratios, which were significantly lower at high phosphorus availability irrespective of the light treatment, both at the beginning and end of the experiment. Light did not increase algal C:P ratios possibly because periphyton growth (and hence C accumulation) was phosphorus-limited in the P− treatments, while the high phosphorus supply in the P+ treatments resulted in algal nutrient saturation regardless of light availability, as observed by Hill et al.^11^. On the other hand, low algal C:P ratios were not associated with improved grazer growth. Snail shell length significantly increased with light and was not affected by phosphorus supply, whereas snail soft body mass was positively affected by light and negatively affected by phosphorus. This outcome contrasts with previous studies in which low algal C:P ratios significantly enhanced snail shell length and/or soft body mass^6,8^. However, Elser et al.^23,24^ demonstrated that high phosphorus supply may be toxic for snail performance and constrain snail growth, which is best achieved at intermediate algal C:P ratios. In the present study, periphyton quantity rather than quality was the main factor promoting snail growth, similarly to results observed by Hill et al.^25^ with the stream gastropod *Elimia clavaeformis*. Moreover, no previous study has specifically tested the effects of phosphorus enrichment on the growth of *A. fluviatilis*. Based on our results, *A. fluviatilis* might have relatively low phosphorus requirements, hence phosphorus enrichment might have constrained rather than stimulated the growth of *A. fluviatilis* soft body mass. Alternatively, snails might have invested the excess phosphorus into reproduction rather than growth. However, for technical reasons, we were not able to properly quantity snail reproduction rates, thus mechanisms of phosphorus allocation in *A. fluviatilis* growth versus reproduction require further investigation.

Despite the positive effect of phosphorus and light on periphyton biomass, we observed an overall decrease in periphyton dry mass in the absence of grazers towards the end of the experiment. This decrease was likely due to the presence of micrograzers in the biofilm, such as chironomid larvae. Nevertheless, micrograzers are commonly found in natural periphyton and did not affect our treatments in a systematic way. Most importantly, micrograzers did not deplete the availability of periphyton for *A. fluviatilis*. At the end of the experiment, the difference in grazed vs. ungrazed periphyton was the strongest in the treatment with high light and high phosphorus supply.

Periphyton community composition was significantly affected by both light and phosphorus at the beginning of the grazing phase, and by phosphorus and grazing at the end of the experiment. Before grazers were added to the flumes, high phosphorus and light availability led to a slight but significant increase in the proportion of diatoms over chlorophytes. Subsequently, during the grazing phase, grazers significantly decreased the proportion of diatoms over chlorophytes only at low phosphorus supply. Several periphyton studies have observed the opposite trend, with diatom abundance decreasing in favour of chlorophytes under phosphorus enrichment^20,26,27^. Nevertheless, in our experimental setup, the water flowing from the river Holtemme was continuously replenishing the diatom community, and phosphorus addition might have favoured the growth of particular diatom species with higher nutrient requirements^28,29^, especially in the absence of grazers. Furthermore, *A. fluviatilis* has previously been described to prefer diatoms to chlorophytes^30^. Although it is unlikely that *A. fluviatilis* could distinguish the two algal groups at cell level in a mixed periphyton community, it is possible that the periphyton taxonomic structure was not homogeneous within the flumes, hence *A. fluviatilis* might have preferentially fed on diatom-dominated periphyton patches, which could explain the overall lower diatom proportion at low phosphorus supply.

Our study demonstrates that the relative strength of bottom-up and top-down control over one another may change over time, and that, in the long run, *A. fluviatilis* individuals are able to adjust their top-down pressure on abundant algal biomass, possibly because snail growth is enhanced by increased food availability. The role of time in shaping food web interactions is particularly relevant in benthic systems, where macrograzers exhibit considerably longer generation times than algae^31^, contrary to planktonic systems^32^. However, nutrient enrichment is overall causing the abundance of aquatic invertebrates to decline^33^, likely due to stoichiometric consumer-resource imbalances, which would eventually lead to a weakened grazing pressure on periphyton. Future studies should address the long-term feedback effects between eutrophication and grazing activity over multiple generations, by testing how phosphorus enrichment would affect not only grazer growth, but also grazer reproduction and survival rates.

Overall, the interaction between bottom-up and top-down control of stream periphyton can be affected by several factors, whose hierarchy may change in the long run. We here demonstrate that the short-term consequences of phosphorus enrichment on grazer feeding behaviour are different from the long-term consequences, resulting in significant fluctuations in the strength of top-down control. Thereby, our results highlight the importance of long-term experiments in the study of resource-grazer interactions.

## Materials and methods

### Experimental set-up

The experiment was performed in the MOBICOS mesocosm facility, a container-based laboratory platform^34^ located by the river Holtemme in Wernigerode, central Germany (51° 49′ 00.7″ N, 10° 43′ 29.26″ E). See Weitere et al.^35^ for detailed water quality data at this station. Each experimental unit consisted of a rectangular flume (62 cm long, 14 cm high and 8 cm wide) constantly supplied with water from the river Holtemme, with a flow rate of 1000 L h^−1^ per flume. The water was filtered by a self-cleaning filter with a mesh size of 50 µm in order to remove larger particles without removing most unicellular organisms. The water level in each flume was 7.5 cm. At the bottom of each flume was a tray containing 30 white ceramic tiles (2.3 × 2.3 cm), disposed in three rows of ten tiles each, and a smaller tray containing nine additional tiles, disposed in three rows of three tiles each. The tiles served as substrates for periphyton growth. Vertical nets were placed at both ends of each flume to prevent grazers from leaving the experimental facility.

The study consisted of a fully factorial experiment, in which two levels of phosphorus supply (high, P+, versus low, P−) were crossed with two levels of light intensity above the flumes (high, L+, versus low, L−) and with grazer presence (G+) and absence (G−), for a total of eight treatments: P+L+G+, P+L+G−, P+L−G+, P+L−G−, P−L+G+, P−L+G−, P−L−G+, and P−L−G−. In the P− treatments, the water flowing in the flumes was kept at ambient P concentration, which was below detection limit (< 3 µg L^−1^ soluble reactive phosphorus, SRP). In the P+ treatments, a concentration of 100 µg P L^−1^, typically observed in eutrophic streams^36^, was achieved in each flume by pumping a constant supply of dissolved KH_2_PO_4_ with a peristaltic pump. The light (PAR) intensity above the flumes, produced by LED lamps, was 111.8 µmol m^−2^ s^−1^ in the L+ treatments and 46.6 µmol m^−2^ s^−1^ in the L− treatments, in a 14:10 h light:dark cycle. In the G+ treatments, eight individuals of the pulmonate snail *A. fluviatilis*, collected in the Holtemme river, were added to each flume. Each of the eight treatments was replicated three times, for a total of 24 flumes.

### Experimental procedure

Natural periphyton was pre-cultivated for two weeks in the experimental flumes, in the respective phosphorus and light treatments (see previous section), from 27 May to 10 June 2019 before adding grazers. On 10 June, three periphyton-covered tiles were selected from each flume for initial sampling. Periphyton was scraped off each tile, homogenised in tap water and filtered onto pre-combusted GF/F glass fibre filters for dry mass, elemental and pigment analyses (see following sections).

On 11 June, eight juvenile *A. fluviatilis* individuals with shell lengths of 2.5–5 mm were added to each G+ flume. The average snail shell length for each flume was between 3.5 and 4.1 mm. Subsequently, the snails were allowed to graze on periphyton for 8 weeks. During this grazing phase, dead or lost snails were replaced with new snails, which were marked with nail polish. Snails that had been added to the experiment after the first 10 days of the grazing phase were not included in the final snail size measurements (see next paragraph). Nevertheless, loss of snails during the experiment was minor, with a maximum of three snails per flume lost after the first 10 days.

On 25 June (day 14 of the grazing phase) and on 17 July (day 36 of the grazing phase), three tiles were selected from each flume for intermediate sampling. Periphyton was scraped off each tile, homogenised in tap water and filtered onto pre-combusted GF/F glass fibre filters for dry mass determination. However, the data collection on 25 June was compromised by a sediment accident that previously occurred in the morning of the same day. A high sediment load was discharged into the Holtemme River due to the cleaning of an upstream basin. Although the water pumped into the MOBICOS facility was cleaned of particles larger than 50 µm with a self-cleaning filter, a large amount of fine sediments entered the experimental facility and shut down the filter for approximately four hours. Due to this immediate impact, data collected on 25 June were excluded from the final analyses. Some sediments remained in the flumes for the rest of the experiment, but periphyton was re-established before the next sampling date. Each experimental flume was affected by the sediments and no systematic effect on specific treatments occurred. Therefore, the event was considered as environmental variability, which is part of the concept of mesocosm experiments, and not as bias to the manipulations on top of the enviromental variability.

On 7 August (day 57 of the grazing phase), the experiment was terminated by removing all grazers from the flumes. Snail shell length was measured with a dial caliper. In addition, snails were freeze-dried and their soft bodies were weighed with a microbalance. The remaining periphyton was scraped off each tile, homogenised in tap water and filtered onto pre-combusted glass fiber filters for dry mass, elemental and pigment analyses.

### Periphyton dry mass and C:P analysis

For dry mass analysis, periphyton samples were filtered onto pre-weighed GF/F glass fibre filters, which were dried at 60 °C for 24 h and weighed with a microbalance to the nearest µg. Periphyton C content was measured with a Vario EL Cube elemental analyser (Elementar Analysensysteme GmbH, Hanau, Germany). For the analysis of periphyton particulate P, filters were transferred to a solution of 9% potassium peroxodisulfate and 0.9% sulphuric acid and heated at 100 °C for one hour in a DigiPREP Block Digestion System (SCP Science, Quebec, Canada). P analysis was subsequently performed with the molybdate-ascorbic acid method^37^ with a DR5000 UV–Vis spectrophotometer (Hach, Düsseldorf, Germany).

### Pigment extraction and analysis

For pigment extraction, filters were transferred into 96% ethanol, left at room temperature for 2 h and stored overnight at − 20 °C. The freezing–thawing cycle was performed twice, and the sample tubes were subsequently placed in an ultrasound bath for 1 min and centrifuged to remove filter residues. The samples were transferred into vials and analyzed via high performance liquid chromatography with a Thermo Scientific UltiMate 3000 HPLC System (Dionex, Thermo Fisher Scientific Corporation, Waltham, MA, USA). Pigments were separated with a reverse phase YMC C30 column. The two solvents used were composed of (A) 45:20:30:5 methanol: acetonitrile:water:ion pair reagent (ammonium acetate + tetrabutylammonium acetate) and (B) 30:50:20 methanol:acetonitrile:ethyl acetate. The flow gradient was the following: 0–4 min: 80% solvent A, 20% solvent B; 35–75 min: 100% solvent B; 77–80 min: 80% solvent A, 20% solvent B. The flow rate was 0.2 ml min^-1^ and the column oven was set at 35 °C.

Pigment measurements were used to estimate the community composition of periphyton with CHEMTAX (version 1.95, Wright and Mackey, Hobart, Australia)^38,39^. The pigment:chlorophyll *a* ratio matrix for oligotrophic environments from Schlüter et al.^21^ was used as input ratio matrix for the P− treatments, whereas the matrix for meso-eutrophic environments^21^ was used for the P+ treatments. From the input matrix, CHEMTAX generated 60 ratio matrices for each treatment. The six matrices (10%) with the lowest residual root mean square were averaged to create a final ratio matrix for each treatment, which was run repeatedly until the ratios and root mean square were stable.

### Statistical analyses

Statistical analyses were performed in R (R Core Team, version 3.6.1, 2019). All data were checked and approved for normal distribution with a Shapiro–Wilk’s test and for homogeneity of variance with a Levene’s test.

Two-way ANOVAs were used to determine the interactive effects of phosphorus and light on periphyton dry mass, C:P ratio and taxonomic composition (measured as diatom abundance) at the beginning of the grazing phase. The interactive effects of phosphorus addition, light and grazing over time on periphyton dry mass during the grazing phase were determined with a linear mixed effects model using phosphorus, light, grazing and time as fixed effects and flume identity as random effect. To analyse the effects of top-down (i.e. grazing) and bottom-up factors (i.e. light and phosphorus) both separately and together over time, the interactions tested in the model were time × grazing, time × phosphorus, time × light, time × phosphorus × light, and time × phosphorus × light × grazing. The individual effects of phosphorus, light and grazing on periphyton dry mass were additionally included in the model.

The interactive effects of phosphorus, light and grazing on periphyton C:P ratio and taxonomic composition at the end of the experiment were determined with three-way ANOVAs. Finally, the interactive effects of phosphorus and light on snail shell length and soft body mass were determined with two-way ANOVAs, where the average shell length and soft body mass for each flume were counted as a replicate. All two-way and three-way ANOVAs were followed by Tukey’s HSD post-hoc tests.

## Data availability

The datasets generated and analysed during the current study are available from the corresponding author on reasonable request.
